# Staying Engaged in Terrorism: Narrative Accounts of Sustaining Participation in Violent Extremism

**DOI:** 10.3389/fpsyg.2020.01338

**Published:** 2020-06-17

**Authors:** Neil Ferguson, James W. McAuley

**Affiliations:** ^1^Department of Psychology, Liverpool Hope University, Liverpool, United Kingdom; ^2^Department of Behavioural and Social Sciences, University of Huddersfield, Huddersfield, United Kingdom

**Keywords:** Northern Ireland, violent extremism, terrorism, radicalization, political violence

## Abstract

Research exploring radicalization pathways and how and why people become involved in terrorism has expanded since the 9/11 attacks. Likewise, over the last decade research exploring de-radicalization and desistence from terrorism has grown and expanded in an attempt to promote exit from extremist or terror groups. However, research studies on how individuals sustain engagement in terrorism and their involvement with extremist organizations, often in the face of great adversity, are absent from the body of research. To address this scarcity of research this study analyzed accounts of engagement in violent extremism produced by Northern Irish loyalist and republican paramilitaries in order to explore how their paramilitary lifestyle, perpetration of acts of political violence and the pressure from countering threats posed by rival groups, and the State security forces impacted on them. The analysis utilized a hybrid of thematic analysis and interpretative phenomenological analysis (IPA). The themes raised through the analysis reflected the psychological, social and economic hardship associated with this lifestyle. The narrative accounts also illustrated psychological changes associated to engagement in violence and from insulation within tightly knit extremist groups. As most of the participants faced incarceration during their paramilitary careers, themes also reflected on the impact imprisonment had on them. The themes explored factors that sustained their involvement, including the role of identity development and identity fusion in sustaining their extremism, the impact of insulated group membership, feelings of efficacy, dehumanization processes, community support, and beliefs in the utility of violence.

## Introduction

While not underplaying the methodological and conceptual problems associated with research exploring terrorism and violent extremism (Silke, 2001; Horgan, 2003; Victoroff, 2005; Schuurman, 2018); research exploring both the routes into violent extremism and processes of radicalization has advanced considerably since the 9/11 attacks. Likewise, the last 10 years have witnessed a growth in research exploring how to bring about an ending of terrorism (Horgan, 2005), through a deeper understanding deradicalization and disengagement from violent extremism at both the individual and organizational level (Ferguson et al., 2015; Altier et al., 2017). However, research focusing on how violent extremists sustain their engagement in violent extremism and deal with the stress and hardships such a lifestyle inevitably entails is missing from these research efforts.

This study aims to begin to fill the gap in this literature by exploring the accounts provided by Northern Irish paramilitaries of their lives after they joined armed groups and the impact this new lifestyle had on themselves and their families. The paramilitaries were from a range of Irish republican and Ulster loyalist groupings, including, the Ulster Defence Association (UDA), Ulster Volunteer Force (UVF), the Red Hand Commando (RHC), Official Irish Republican Army (OIRA), Provisional Irish Republican Army (PIRA), and the Irish National Liberation Army (INLA). These narrative accounts have been analyzed through a combination of Interpretative Phenomenological Analysis (IPA; Smith, 1995, 1996) and Thematic Analysis (Braun and Clarke, 2006) to understand how the participants interpreted and made sense of their time as active paramilitaries, the factors that sustained their involvement in violent extremism and the impact it had.

Research within terrorism studies is somewhat limited, and has been slow to explore this aspect of the “arc of terrorism,” or the “terrorist lifecycle” (Horgan and Taylor, 2011; Horgan, 2017). Nevertheless, some of the most popular radicalization models touch on factors that could be involved to sustaining extremism beyond early encounters with radical groups and use of violence for seemingly political or ideological reasons. Due to the proliferation of radicalization models, (for example, the most cited models include: Borum, 2003, 2004; Moghaddam, 2005, 2007; Taarnby, 2005; Wiktorowicz, 2005; Taylor and Horgan, 2006; Gill, 2007; Precht, 2007; Silber and Bhatt, 2007; McCauley and Moskalenko, 2008; Sageman, 2008; Dalgaard-Nielsen, 2010; Sinai, 2012; Kruglanski et al., 2014) and the considerable overlap across models (Jensen et al., 2016), this article will restrict its focus to the three most widely cited models (Moghaddam, 2005; Taylor and Horgan, 2006; McCauley and Moskalenko, 2008).

McCauley and Moskalenko (2008) noted how Irish republicans’ sustained long-term involvement through interdependence and devotion to the wider group. While they also argue that immersion in a threatened compliant like-minded group, should push members toward ever-riskier courses of action. Moghaddam (2005) also supports much of these ideas, arguing that terror groups use isolation, devotion and ingroup pressures to create a moral disengagement from the mainstream in order to foster a moral engagement with the ideology and norms of the extremist group in the face of external threat. Taylor and Horgan (2006) further describe how involvement with the group would involve practical training and political or ideological education or exposure, in order for the militant to be able to understand their violence or actions on behalf of the movement within organizational norms. Thereby, strengthening involvement while also providing meaning and direction to the militant’s actions, leading to their deepening institutionalization within the group and a merging of their personal and group identity.

Research exploring the impact of engagement in armed violence is also available from other sources. Firstly, from studies of soldiers who have been deployed to fight in conflicts across the globe, generally as part of the “war on terror,” which like research in terrorism studies has flourished since 9/11 (Engen, 2008; Cigrang et al., 2014; Russell et al., 2014; Cabera et al., 2016). Secondly, through research on child combatants (Hermenau et al., 2013), and finally, and most pertinently to this study, research on the impact of imprisonment for former combatants in Northern Ireland (Jameison et al., 2010; Shirlow et al., 2010; Ferguson, 2014).

Research exploring the experiences of both regular soldiers and irregular militias, including those incorporating child soldiers in their ranks illustrate some of the problems faced by people deployed in combat who engage in organized killing. One key problem is that, as Grossman (1996) and Marshall (2000) observed, the average “normal” soldier has an inner resistance to killing fellow humans, even enemy humans and to overcome this resistance, soldiers have to be desensitized, and conditioned to kill through their military training. However, even after being trained to kill, Grossman (1996) argues that for soldiers who engage in combat, the number of psychiatric casualties will outnumber the physical.

Indeed, he argues that a large proportion of combat troops will have significant negative psychiatric outcomes, such as physical and mental exhaustion, dissociation from reality, anxiety, depression, somatic symptoms, OCD, etc.

While Grossman (1996) and Marshall (2000) are not without their critics (Ghiglieri, 1999; Chambers, 2003; Engen, 2008) the evidence that engagement in combat can have negative physical and psychological impact is overwhelming (Smith et al., 2008). Studies have consistently demonstrated problematic mental health, alcohol and substance use, relationship health, readjustment problems, increased anger and aggression among soldiers and child soldiers post-combat (Keyes et al., 2012; Hermenau et al., 2013; Cigrang et al., 2014; Russell et al., 2014; Cabera et al., 2016). Furthermore, McNair (2002) has demonstrated higher rates of PTSD amongst those who did the actual killing, especially amongst those who killed civilians, in comparison to others who just observed killing. Based on these studies the expectation is that the noted psychological problems would occur amongst violent extremists and Northern Irish paramilitaries who engaged in acts of ideological or political violence in addition to members of regular armies. Indeed, as these individuals operated outside the law, it would have been difficult, if not impossible to receive therapeutic support for psychological problems caused by their own violence. The implications of this is that there may be a disproportionate number of former paramilitaries with undiagnosed problematic mental health (Ferguson et al., 2010).

Research with former politically motivated prisoners and former combatant groups from Northern Ireland consistently demonstrate the negative impact engagement in political violence has for the perpetrator (Shirlow and McEvoy, 2008; Jameison et al., 2010; Shirlow et al., 2010; Shirlow, 2014). For example, while one percent of the Northern Irish population were fatalities during the Troubles, 45% of former paramilitary prisoners had a relative killed (Shirlow and McEvoy, 2008). Former paramilitary prisoners have high rates of suicide (Shirlow, 2014), reflected in 38% of former loyalist prisoners reporting that there were times when to did not want to “go on living” post release (Jameison et al., 2010). While rates of PTSD in Northern Ireland are higher than in other comparative countries (Bunting et al., 2012) at nine percent, indicators of PTSD, such as experiencing intrusive memories and dreams were reported by 51% of former paramilitary prisoners (Jameison et al., 2010). Similarly, rates of mental illness (41%), use of anxiety and depression medication (41%), hazardous drinking (68%), and alcohol dependency (53%) were up to four times the regional or national averages amongst former paramilitary prisoners (Jameison et al., 2010).

Research exploring engagement in social movements or in collective action have demonstrated the importance of identity in predicting (Van Zomeren et al., 2008), fuelling (van Stekelenburg et al., 2010), and sustaining engagement with social movements (Klandermans, 1984). Likewise, research exploring disengagement and deradicalization processes demonstrate the importance of identity in stimulating disengagement from violence (Ferguson et al., 2015, 2018; Ferguson, 2016; Raets, 2017). Research has also demonstrated that once individuals become active members their identities can be strengthened (Vestergren et al., 2016) and they can become fused with the group identity (Swann et al., 2012), cut themselves off from external groups and become increasingly bound to the group aims and norms (della Porta, 1995). Given these patterns, we would expect that prolonged engagement in paramilitary activity would affect identity and strengthen bonds to the armed group and the wider community that support these groups.

A review of these areas of research suggests that engaging in a prolonged period of violent extremism will have negative economic, physical, psychological and social impacts on former Northern Irish combatants, and that this engagement will further impact on their identity, attitude and behavior (McAuley et al., 2010). This current study will explore the narrative accounts of Northern Irish militants who engaged in politically motivated violence during the conflict in Northern Ireland in order to explore the factors that sustained this violent extremism, the impact that living this lifestyle had on the combatants and how it manipulated their identity, cognition and behavior. In doing this, the study will provide insights on an aspect of the violent extremist lifecycle which has been absent from analysis.

## Materials and Methods

### Participants and Data Collection

All the participants (*n* = 110) were members or former members of Northern Irish paramilitary groups. The sample was comprised of predominantly male (*n* = 102) ex-prisoners (*n* = 105) and included both loyalist (RHC, *n* = 3; UDA, *n* = 20; UVF, *n* = 48) and republican (INLA, *n* = 9; IRA, *n* = 30) paramilitaries^1^. All the participants had been interviewed by research teams involving the first or second author as part of a variety of research projects conducted since the 1990s. When interviewed the participants were a combination of active and former paramilitary combatants, and the sample contained a blend of both leadership and “rank and file” members. However, as the original interview transcripts had been previously anonymized it was not possible to provide exact data on age, the composition of rank, length of service or membership status of the participants. The original research projects had received ethical approval from an author’s host university and this study received ethical approval from the first author’s university and the [Centre for Research and Evidence on Security Threats (CREST)] at the (Lancaster University).

All of the interviews had been semi-structured, conducted face-to-face and lasted from 30 min to over 3 h, the interviews had focused on issues related to the participants’ experiences as combatants during the Troubles and their thoughts around various issues related to the conflict, their paramilitary activity, imprisonment and the peace process. All interviews were transcribed and anonymized to maintain participant anonymity and confidentiality. In addition, to enhance the participants’ anonymity, the Ethics committee based at (CREST) requested that the quotes presented should not be in the interviewees own words, thus all quotes presented in the article have been paraphrased from the original transcripts. In order to secure coherence and the integrity of the paraphrased quotes both authors reviewed the original and paraphrased versions to ensure the meaning expressed in both versions was consistent.

### Data Analysis

The data was analyzed inductively (Patton, 1990) without trying to fit with previous conceptualizations or research in this area. The analysis followed processes and principles common to thematic analysis (Braun and Clarke, 2006) and guided by principles shared with the Interpretative Phenomenological Approach (IPA; Smith, 1995; Smith et al., 1999) as the research aimed to make sense of the participants’ lived experiences.

The first author began the analysis by reading and re-reading the anonymized transcripts, noting ideas and while focusing principally on parts of the transcripts were the participant explored their engagement in politically motivated violence and with paramilitary groups. Following this initial stage, the first author then began to re-analyze the transcripts line-by-line and note aspects of the interview with psychological significance in the left-hand margin. These raised codes were couched in the participants’ narrative and driven by the data they had individually produced. In the third and final stage of analysis, the first author returned to the transcripts and began to collate and refine the different codes into themes, super-ordinate themes and sub-themes. During this process, the first author constantly returned to the original transcripts to confirm that the themes reflected the same meaning across the participants’ accounts.

Once the first author completed this three stage analysis, the second author independently examined the transcripts, summary documents, codes, and themes in line with Yin (1989) to provide an audit in order to enhance the qualitative validity of the analysis and themes (see Yardley, 2000). The second author’s audit assessed the coherence of the themes, their rigor that they represented the data, were sensitive to the context and grounded in the participants’ accounts. While no software can actually analyze qualitative data (Ose, 2016), due to the large dataset MS Word and MS Excel were utilized (Meyer and Avery, 2009) for data management and to support the analysis process.

## Results

The six themes raised from the data related to life after joining a paramilitary group and how this lifestyle affected the participants. The first theme “moral ambiguity, dehumanization and isolation” explores the psychological changes the interviewees reported that they underwent due to the pressures of being a militant in a closed extremist group. The second theme “all-encompassing identity” demonstrates how being a militant becomes a central feature of the paramilitary’s sense of who they are, playing an important role in both sustaining engagement in morally ambiguous actions and serving as a protective factor. The third theme examines increases in a “sense of purpose and efficacy” that resulted in taking a stand. The fourth theme explores how the participants’ believed that “violence effectively brings political change” and brought benefits to their wider communities. The fifth theme acknowledges the importance of “community support” in sustaining the participant and their organization in armed actions. The final theme “imprisonment, radicalization and strategic development” explores how prison could be transformative, allowing the imagining of different futures and paths.

### Theme 1: Moral Ambiguity, Dehumanization and Isolation

The majority of the Northern Irish working class live in communities segregated along ethno-political lines, which has a significant impact on worldviews and reasoning (Ferguson and McKeown, 2016). Once people commit to these armed groups they begin to socialize within even narrower groups of loyalist or republican extremists inside these divided and insulated communities. Then as they become operatives in the conflict, and are forced to live with the pressures and stressors associated with this lifestyle, they socialize within even smaller circles of likeminded paramilitarists who they felt they could trust. As they are also under constant surveillance and threat, they become more paranoid, obsessive and insulated from people outside the extremist group.

When you give your life to something. And it’s not just jail, people go on about jail as a big loss, but the second you walk into the room and say “I stand up as a member of the IRA until the second the struggle is over,” you have no life. You literally become obsessive. You trust nobody. Every detail has to be examined. You have to cross every “t” and dot every “i” and check every single thing (T52, PIRA, Belfast).

These insulated groups amplify ingroup identities (see “Theme two”), beliefs and biases, while hardening stereotypes and negative enemy images of the rival armed groups and the wider community from which they are drawn. This fuels moral disengagement (Bandura, 2004) and the development of dehumanizing perceptions of the other, which are necessary to allow members of the armed groups to justify and engage in politically motivated murder (Grossman, 1996).

Anyone on the nationalist side whose lips moved was a legitimate target. I don’t believe that now, but back then I believed that. I watched Bobby Sands^2^ funeral and I saw a hundred thousand people in the graveyard in west Belfast. Up till then I was only prepared to shoot dead republicans. But after that day I viewed all nationalists and all Roman Catholics in west Belfast as legitimate targets, because I felt they were all supporting the armed struggle (T94, UVF, Belfast).

You just didn’t think about it. When you’re firing at soldiers or firing a grenade, or whatever you were doing, you didn’t see the target as a person. You seen it as a symbol of the state. You never thought of it, of whether they had kids or whether they were 18 years of age – the same age as you (T53, PIRA).

This isolation, coupled with a stressful and threatening environment and reliance on negative stereotypes of the opposition, creates risky shift (Stoner, 1961) or Groupthink like pressures (Janis, 1982) which contribute to risky decision making and morally ambiguous decisions based on a poor analysis of the situation, as illustrated by this UVF member:

The idea was by taking out one of their top brigadiers, that it would end the feud. And which, unfortunately, it never does (T22, UVF, Belfast).

In this stressful and insulated environment, the paramilitaries focus on short-term strategy, engaging the opposition, reacting to their attacks and fighting the war, there is little room for long-term strategic or political analysis. It is not until they are removed from the conflict, usually through incarceration, that they begin to develop a more coherent, and sophisticated strategy beyond the “tit for tat” sectarian violence that fuelled their initial engagement (see Ferguson and McAuley, 2019). This evolution in political awareness through incarceration and/or longer-term engagement with the organization is further discussed in theme six.

### Theme 2: All-Encompassing Identity

It is clear from quotes in the earlier theme that the participants’ activism pervades all aspects of life and they have sustained their involvement in the military, social-welfare or political activities of these proscribed armed organizations and their political affiliates in some cases over decades, and in face of some significant challenges and existential threats to themselves and their families. Indeed, experiencing these challenges, coupled with ingroup insulation increasing cohesion, was key to bonding their group and personal identities and fuelling their agency (Swann et al., 2012; Doosje et al., 2016). From the interviews, it was clear that having a strong and all-encompassing activist identity was key to sustaining these challenging years of activism through the conflict and into the peace.

I’m a loyalist. That’s why I’m in politics and the UPRG (Ulster Political Research Group). I believe, I won the war militarily, they didn’t deter me. And when I came out of prison, I thought what am I going to do – sit with my feet up and say to my grandkids or my children “oh I done this during the war, I done that during the war” or go and face them [Sinn Fein]. And we have taken that challenge up and the more we do face them and challenge them the more confident I get (T82, UDA, Mid-Ulster).

In line with previous research (Swann et al., 2012; Hogg and Adelman, 2013; Ferguson and Binks, 2015; McDonald, 2018), once the individual becomes a member of the group they are instilled with the group norms and feel a reduction of uncertainty. These changes are part of the process that is necessary to overcome the cognitive and emotional barriers to killing (Grossman, 1996; Hoover Green, 2016). This risky groupthink style thinking gives their violent or criminal actions and the morally ambiguous activities of their organization, a sense of moral clarity and provides the required zeal to sustain their involvement.

People kept their spirits high, people knew where they belonged, and it was easy to do what you did. It was clear what you were doing (T37, PIRA, Female).

This sense of comradeship and *esprit de corps* is further developed when the paramilitary becomes incarcerated. The time spent in segregated prison wings with likeminded paramilitarists developed strong bonds of friendship bonds that would further sustain their engagement on their release.

Because of prison I have gained a lot. Obviously, I’ve lost a lot, but I’ve gained a lot as well. Like the friendship, there are strong bonds that are created through the conflict, for me that’s a personal gain. Again, a lot of losses, a lot of friends were killed, a lot went to prison, and they spent a long time in prison (T31, INLA, West Tyrone).

These processes of increasing isolation and corresponding attachment and identification to comrades and the wider organization share similarities with the predictions from identity fusion theory (Swann et al., 2012) and the devoted actor model (Atran et al., 2014). Swann et al. (2012) predict identity fusion to arise when group members experience visceral feelings of oneness with their group until the point where the personal and social selves fuse into one singular identity. These fused group members form kin-like attachments with fellow fighters and become devoted to the collective. This also encourages antisocial pro-group behaviors, such as fighting and dying for their country (Swann et al., 2009; Gómez et al., 2011; Whitehouse et al., 2014). These processes are clearly at work in the narratives produced by the interviewees, for example:

It stripped me completely from being a father, husband, brother, son, it stripped me of that identity, of what I was to other people, I became this person who was completely focused on the other side. Now, in hindsight, my biggest regret is quite simply that I put something before a wife and daughter (T26, UVF, North Antrim).

These processes of identification provide a lens from which the protagonist understands the conflict and makes sense of their personal actions in the conflict. Group membership can be important in mediating the stress and trauma that is associated with involvement in political violence (Muldoon et al., 2016). Therefore, this amplification or fusion of identity should additionally offer protection from the stress and trauma they encounter or create for themselves through their violence, sustaining the militants in their extremist careers.

### Theme 3: Sense of Purpose and Efficacy

This collective identity fed into a sense of purpose, generating feelings of empowerment and efficacy in the face of an evil dehumanized foe.

Obviously when you do things that you normally won’t consider you’d be capable of doing and facing the possibility of death and jail for doing them. Then that’s obviously a negative, but you felt that your country was under siege and that you were doing something about it. In the middle of that came the Ulster Workers Strike, and that was the closest thing that I could find to what people might term the “Blitz spirit,” were everyone stuck together. We all felt proud that we were serving our country, because the security forces at that time didn’t seem to be making any progress, in terms of taking the fight to the IRA (T25, UVF, Belfast).

Research exploring domestic (Decou et al., 2015) and occupational violence (Yao et al., 2014) have demonstrated that increased self-efficacy is related with better coping with experiences of violence. This suggests that while higher efficacy will clearly be important in sustaining extremism, it may also have a protective function that helps sustain engagement by mediating the impact of trauma, as suggested by this IRA volunteer:

When it came to my generation we said, “That’s it. We’re standing up and we’re not taking any more.” But, it was for the sake that all our children, yet unborn would have a better life. So, I was prepared to sacrifice myself quite truthfully. And I’m lucky. I’m one of the lucky ones that survived it. I was shot twice, wounded twice and friends of mine were shot dead. They’re in the cemetery. And, God love them (T15, OIRA, Derry).

### Theme 4: Violence Effectively Brings Political Change

While many of the interviewees acknowledged that some of their counterparts had joined paramilitary groups for their own personal status or financial benefit; none of the interviewees gave this as a reason for their own involvement. Indeed, many discussed how their membership and imprisonment had led to significant financial hardship for them and their families. Instead, the former combatants saw the benefits of their activism in terms of community, rather than personal gains. They believed their activism and the associated risks they had taken had kept their community safe, helped initiate the peace process or brought about a positive change in the social conditions of the wider community they originated from.

I think overall the position of nationalism, without a shadow of a doubt, has been enhanced greatly through the armed conflict. Prior to 1969 we had discrimination and even though sectarianism is still rampant and rife, it’s more subtle than it was back then. The armed struggle has put a great confidence in the nationalist people and a great confidence in republicans. They haven’t achieved their objectives of a united Ireland, but they’ve achieved a lot of stuff. They might not have achieved total equality but there’s no doubt it has improved (T31, INLA, West Tyrone).

A key aspect of this belief was that the actions of the armed group they belonged to, and thus their own violence, had improved or at least maintained the position of their community in face of threats from the other community or the British Government. Many participants believed that the violence they had employed was effective in bringing change, or forcing the opponent to seek a political compromise. Republicans drew widely on the belief that violence had brought about changes in Irish society, which otherwise would not have been achieved, for loyalists it was their violence that had brought the republican movement to its knees, eventually to settle for a ceasefire. Witness the following:

I’ll believe to the day I die that our strategy from the mid 1980’s brought the republican movement to their knees. We were out-killing them and out-gunning them. We were hitting the right people. Regardless of what they say, we knew who we were hitting. And even when I was in the jail. I knew we were winning because I could see it on their faces. You just seen it when they came in. You knew they were a defeated people. I will take to my grave that we won the war. Militarily, we won the war (T82, UDA, Mid-Ulster).

At times this belief in the effectiveness of violence, could be quite nuanced, so while there was a widespread recognition that violence had resulted in positive social and political changes, there was also a general understanding it was not a solution to the greater political problems faced in Ireland. They also widely acknowledged that the sectarian nature of the violence created barriers that had made a peaceful solution to the conflict more difficult to obtain.

I think personally, it was too little gain for too much lost. The loss wasn’t worth the gain. You could have had this 30 odd years ago with Sunningdale^3^, with just a few variations. There is too much pain and too must loss for too little gain. Sunningdale for slow learners, as Seamus Mallon^4^ said (T28, INLA, Belfast).

### Theme 5: Community Support

In addition to strengthened identity, and increased beliefs in personal efficacy sustaining involvement, there was an understanding that the paramilitary groups were only able to exist and function with the wider support of the communities they were embedded in. Thus, community support was essential to maintaining the organizations and their violent campaigns.

In my community we couldn’t have worked or operated without them. They were a vital cog in the war. They provided us with safe houses. They provided us with information, they provided us with alibis. We couldn’t operate without them, we couldn’t have worked without them (T27, UVF, North Antrim).

It is clear that both republican and loyalist armed groups had the support of at least a considerable minority of their respective community (Hayes and McAllister, 2005). While this support was fundamentally taken for granted by the republican interviewees, for a large proportion of the loyalist participants, there was an acknowledgment that this support could be, in the words of one former UVF member, “mercurial,” and that the working class Protestant communities were less supportive of loyalist paramilitary groups than their republican counterparts. With the level of support offered reflecting the severity of the threat the wider Protestant community perceived to be emanating from republicans or a “treacherous” British Government (see Reed, 2015; McAuley, 2016).

### Theme 6: Imprisonment, Radicalization and Strategic Development

While initial engagement in political violence was reactive (Ferguson and McAuley, 2019), paramilitary careers are not static, they are dynamic and continuously molded by life events, organizational changes, and the impact of outside forces. Deeper and sustained involvement is marked by an increased engagement with the organization, its members, norms and ideologies (see “Theme two”). Initial violence is now interrupted through sophisticated ideological lens that have been developed through introspection and educational opportunities, primarily provided through imprisonment:

Someone once said “isolation breeds introspection,” certainly prison was an opportunity, however, unwelcome, to consider where we have been, and more importantly, where we are going. For some prison was a rude awakening, serving life on the basis of “kill all taigs” is little comfort when faced with the reality of losing your freedom, family and future. There has to be more than crude hatred. I listened carefully to what my comrades said. I listened to the politicians. I considered the role of the paramilitaries. I endorsed or rejected views which I felt were outdated or impractical. In my opinion, prison enabled men like me, to develop ideas which provided the groundwork for a stable and pluralist society (T95, UVF, Belfast).

Exposure to prison education and having the space for reflection was a key period in their militant career. It provided the opportunity for flexible deliberative System 2 thinking (Kahnerman, 2011) and prompted them to consider the nature of the conflict, and what was achievable in the long-term. Prison offered access to resources that were scarce in the working class republican and loyalist communities outside prison. For example, they held discussions with ideologs, returned to education, and developed their thinking beyond the intuitive System 1 thinking that had been key in much of the initial violence. For some they had the opportunity to participate in dialogue with representatives of oppositional groups, which provided opportunities to focus on contradictory viewpoints and challenge some of the dehumanized zero sum assumptions that they held prior to incarceration.

I became involved with the UVF when I was fourteen. I would have had a strong sense of being an Ulsterman or being British because of my father, and him serving in the war. I would have believed that God was Protestant, so believed that God was on our side and that we were right. Prison changed that, contact with republicans, those human relationships and conversations, started to breakdown that demonization that the enemy were all bad, that they were just evil, and that was that. Conversations in that prison context as humans made me realize that at the end of the day we are all the same (T90, UVF, Mid-Ulster).

To some degree, prison was a radicalizing process, in that it provided the space to develop ideological and political acumen that was absent at the beginning of the paramilitary career, as discussed by this INLA volunteer.

Before you go to prison, unless you’re actually very astute politically, all you really think about is going out and doing the business. But once you get in there you have the time to sit back and talk about things and realize that the only way of going forward at the minute is politics. You can’t keep killing people forever (T35, INLA, Derry).

Incarceration was important in providing violent militants with new lens to review the conflict. This ideological turn then provided the grounding for many to begin a personal transformation away from using violence. A journey, which would push some toward seeking conflict transformation, rather than military victory.

Prison was very important because that’s where the embryo of the PUP [the Progressive Unionist Party] was actually created. That’s where the embryo of the first peace initiatives were found because prisoners went on to question “why am I in here?” You have to ask yourself, “how did I end up shooting somebody because it’s not in my nature?” If I had of been born elsewhere I would never have seen the inside of a prison cell. So, you start to question what society can create a situation where people go out and blow people up or shoot people dead. When just a year beforehand they would never have thought of doing any such thing. When you start to question those assumptions that’s when you start to develop your thoughts and ideas (T77, RHC, Belfast).

While prison was radicalizing, in that, it involved an incremental commitment to political ideology (Horgan and Braddock, 2010). Incarceration also led to a move away from violent extremism as the sole method of bringing political change, as witnessed in the rise of the PUP and UDP (Ulster Democratic Party) and the move to the “armalite and ballot box” strategy of Sinn Fein and PIRA. This strategic turn allowed seasoned paramilitarists the opportunity to sustain their career via alternative pathways into community and politically focused avenues, routes that would have been unimaginable at the inception of their paramilitary careers.

## Discussion

The findings presented provide unique insights into the impact joining an armed group and engaging in violent extremism has for the extremist. It is clear from the narrative accounts the intense pressure and hardship a person and their family face when someone embarks on this militant lifestyle. Many of these stressors and hardships cut across the themes and relate to the psychological, social, economic, familial, and physical harm presented in the findings from combat veterans (Cigrang et al., 2014; Cabera et al., 2016). However, the accounts also demonstrate a less understood level of loss, in terms of how this lifestyle can result in the extremist losing their sense of self, control over their own lives, their sense of conscience and their moral compass in order to fuse with the organizational goals and participate in violence and killing (Bandura, 2004; Swann et al., 2012). Accounts of these harms drawn from actual militants are largely absent from the terrorism studies literature, and require further investigation.

In parallel with these negative consequences and losses, being a paramilitary led to an amplification in radical views and beliefs in the utility of violence. It strengthened feelings of empowerment and self-efficacy, while increasing the use of dehumanization and bias. These processes, developed through insulated contact between likeminded militants subjected to threat (Stoner, 1961; Janis, 1982), are necessary to sustain a militant lifestyle and buttress the militant against the pressures they face (Yao et al., 2014).

These narrative accounts clearly have synergy with the findings and predictions from identity fusion theory (Swann et al., 2012) and the devoted actor model (Atran et al., 2014). It is clear in the themes that engagement with the armed group leads to a shift in identity salience with personal identities becoming more fused (Swann et al., 2009) to the collective. These kin-like bonds and feelings of esprit de’ corps are important for sustaining involvement and protecting the militant from both the external pressure and the self-harm caused by their actions. Findings reflective of the well-established links between health and identity in the face of political conflict (e.g., Muldoon et al., 2016). These presented accounts of engagement also reflect predictions from models of radicalization and the wider social movement literature (della Porta, 1995; Moghaddam, 2005; Taylor and Horgan, 2006; McCauley and Moskalenko, 2008; Vestergren et al., 2016). Demonstrating how devotion to the group and its members, under the conditions of isolation, threat and exposure to ideological education, develop activist identities, promote radicalization and sustain involvement with extremist groups even in face of grave adversity.

There was an understanding that for most militants a career as a paramilitary would inevitably lead to imprisonment or premature death, clearly two very negative life events. However, somewhat counter-intuitively the former prisoners tended to reflect on prison as a positive and transformational opportunity. The positive consequences of imprisonment reported here resonate with research on post-traumatic growth (Tedeschi and Calhoun, 2004) amongst long-term “ordinary” prison samples (Van Ginneken, 2016), prisoners of war and political prisoners (Salo et al., 2005; Solomon and Dekel, 2007). For these participants this transformational growth provided further support for their activism, while the educational and political knowledge they developed in prison was central to the transformation of the wider organization and the group’s long-term political strategy. A transformation that would eventually remove organized violence from the group’s strategic repertoire and see all the armed groups sampled in this study participate in the Northern Irish peace process to varying degrees.

These findings demonstrate the need to understand the terrorist or extremist lifestyle beyond the study of initial engagement or disengagement, the insights provided by the participants will help to develop a greater understanding of the processes involved in both maintaining and challenging violent extremism. They provide challenges for those involved in countering violent extremism and terrorism, on how to promote attitudes, identities and beliefs that will hasten to move individuals away from violence and/or extremist organizations toward desistence from violence and terror tactics. The findings also suggest synergies with other areas of study, such post-traumatic growth (Tedeschi and Calhoun, 2004) which have not received substantial attention in relation to violent extremism. In addition, to supporting theories which have made much more significant contributions, such as social movement approaches (Klandermans, 1984; van Stekelenburg et al., 2010; Vestergren et al., 2016), identity fusion (Swann et al., 2009, 2012), and social identity approaches (Van Zomeren et al., 2008).

All conflicts and armed groups are unique. Therefore, the lessons learned from an exploration of one context do not necessarily translate to other contexts. However, the themes raised through this analysis do show similarity to models of involvement in violent extremism (e.g., Moghaddam, 2005; Taylor and Horgan, 2006; McCauley and Moskalenko, 2008; Doosje et al., 2016). Therefore, the findings may provide a useful framework from which to explore how militants in other clandestine armed groups operating in different contexts sustain their involvement in violent extremism in face of external threats and the negative impact of this life path.

As with the vast majority of research on activism and engagement with violent extremism, (see Vestergren et al., 2016 for as discussion of these issues) this research relies on cross-sectional retrospective accounts collected at different time points, within the specific context of Northern Ireland. To remedy this weakness future research should attempt to be prospective and capture longitudinal data. Although we acknowledge, given nature of the research area, the ability to identify potential extremists and to collect pre-engagement data would be problematic, and pose demanding ethical dilemmas.

## Data Availability Statement

The datasets generated for this study will not be made publicly available. The data are taken from anonymized interviews of former combatants and prisoners from Northern Ireland, due to sensitivities around the content they are not publicly available. In addition neither author have the permission of the original participants to share this data in its compete state. Requests to access the datasets should be addressed to the corresponding author.

## Ethics Statement

The studies involving human participants were reviewed and approved by the Faculty of Sciences Ethical Committee at Liverpool Hope University and Centre for Research and Evidence on Security Threats (CREST) at the Lancaster University. The patients/participants provided their written informed consent to participate in this study.

## Author Contributions

NF was involved in collecting the original data, constructing the data set, analyzing the interview transcripts, and writing up the manuscript. JM was involved in collecting the original data, constructing the data set, auditing the analysis, and in the write up the manuscript.

## Conflict of Interest

The authors declare that the research was conducted in the absence of any commercial or financial relationships that could be construed as a potential conflict of interest.

## Footnotes

1 Other data from this population was also analyzed in Ferguson and McAuley (2019).

2 Bobby Sands was a PIRA volunteer who led, and died on hunger strike while serving a sentence for possession of firearms at HMP Maze in 1981. We was also elected as an MP while on hunger strike, dying less than a month after his election.

3 The Sunningdale Agreement was a failed attempt to bring together a power-sharing government in Northern Ireland in 1973.

4 Seamus Mallon was the deputy leader of the Social Democratic Labour Party (SDLP) and served as deputy First Minister of Northern Ireland from 1998 to 2001.
